# *In-vitro* susceptibility and *ex-vivo* evaluation of macrocyclic lactone endectocides sub-lethal concentrations against *Plasmodium vivax* oocyst development in *Anopheles arabiensis*

**DOI:** 10.1186/s12936-024-04845-x

**Published:** 2024-01-18

**Authors:** Gemechu Zeleke, Luc Duchateau, Delenasaw Yewhalaw, Sultan Suleman, Mathias Devreese

**Affiliations:** 1 Department of Pathobiology, Pharmacology and Zoological Medicine, Faculty of Veterinary Medicine, Ghent University, Salisburylaan 133, Merelbeke, Belgium; 2 Jimma University Laboratory of Drug Quality (JuLaDQ), and School of Pharmacy, Institute of Health, Jimma University, Jimma, Ethiopia; 3 Biometrics Research Center, Faculty of Veterinary Medicine, Ghent University, Salisburylaan 133, Merelbeke, Belgium; 4 School of Medical Laboratory Sciences, Institute of Health, Jimma University, Jimma, Ethiopia

**Keywords:** Membrane feeding, *Plasmodium vivax*, *Oocyst*, Sublethal concentrations, *Anopheles arabiensis*

## Abstract

**Background:**

Asymptomatic malaria transmission has become a public health concern across malaria-endemic Africa including Ethiopia. Specifically, *Plasmodium vivax* is more efficient at transmitting earlier in the infection and at lower densities than *Plasmodium falciparum*. Consequently, a greater proportion of individuals infected with *P. vivax* can transmit without detectable gametocytaemia. Mass treatment of livestock with macrocyclic lactones (MLs), e.g., ivermectin and doramectin, was suggested as a complementary malaria vector tool because of their insecticidal effects. However, the effects of MLs on *P. vivax* in *Anopheles arabiensis* has not yet been fully explored. Hence, comparative *in-vitro* susceptibility and *ex-vivo* studies were conducted to evaluate the effects of ivermectin, doramectin and moxidectin sub-lethal concentrations on *P. vivax* oocyst development in *An. arabiensis*.

**Methods:**

The 7-day sub-lethal concentrations of 25% (LC_25_) and 5% (LC_5_) were determined from *in-vitro* susceptibility tests on female *An. arabiensis* in Hemotek® membrane feeding assay. Next, an *ex-vivo* study was conducted using *P. vivax* gametocytes infected patient’s blood spiked with the LC_25_ and LC_5_ of the MLs. At 7-days post-feeding, each mosquito was dissected under a dissection stereo microscope, stained with 0.5% (w/v) mercurochrome solution, and examined for the presence of *P. vivax* oocysts. Statistical analysis was based on a generalized mixed model with binomially distributed error terms.

**Results:**

A 7-day lethal concentration of 25% (LC_25_, in ng/mL) of 7.1 (95% CI: [6.3;8.0]), 20.0 (95%CI:[17.8;22.5]) and 794.3 (95%CI:[716.4;1516.3]) were obtained for ivermectin, doramectin and moxidectin, respectively. Similarly, a lethal concentration of 5% (LC_5_, in ng/mL) of 0.6 (95% CI: [0.5;0.7]), 1.8 (95% CI:[1.6;2.0]) and 53.7 (95% CI:[ 48.4;102.5]) were obtained respectively for ivermectin, doramectin and moxidectin. The oocyst prevalence in treatment and control groups did not differ significantly (p > 0.05) from each other. Therefore, no direct effect of ML endectocides on *P. vivax* infection in *An. arabiensis* mosquitoes was observed at the sub-lethal concentration (LC25 and LC5).

**Conclusions:**

The effects of ivermectin and doramectin on malaria parasite is more likely via indirect effects, particularly by reducing the vectors lifespan and causing mortality before completing the parasite’s sporogony cycle or reducing their vector capacity as it affects the locomotor activity of the mosquito.

**Supplementary Information:**

The online version contains supplementary material available at 10.1186/s12936-024-04845-x.

## Background

Malaria is a life-threatening disease, especially in children, with a high prevalence in sub-Saharan Africa. Globally, an estimated 247 million malaria cases and 619,000 malaria deaths were reported in 2021, of which more than 95% of the cases and deaths were in Africa [1]. Interventions targeting both the vector and the parasite are vital for malaria control and prevention. Long-lasting insecticidal nets (LLINs) and indoor residual spraying (IRS) are the principal malaria vector control tools which have been in use for decades [2, 3]. Together with the current growing population number in malaria endemic countries, the increasing price of the malaria control tools, such as LLINs and IRS creates a challenge on their universal access and use [4, 5]. In addition, the widespread insecticide resistance and behavioral adaptations (a shift from indoor to outdoor feeding behaviour) by the *Anopheles* vectors threatens the effectiveness of the current vector control tools [6–9]. Currently, neither LLINs nor IRS are suited to target outdoor biting mosquitoes and reduce residual malaria transmission in different areas of Africa [10]. Consequently, most African countries are still far from achieving malaria elimination. Therefore, further progress towards malaria elimination requires complementary efforts of greater funding, universal access and use of the available control tools, and new more cost-effective tools.

*Anopheles arabiensis* and *Anopheles gambiae *sensu stricto (*s.s*.) are the common malaria vectors in Africa, which are susceptible to *Plasmodium vivax* and *Plasmodium falciparum* infections [11, 12]. The sibling species can have different levels of vector competence and vectorial capacity. *Anopheles gambiae s.s.* is known for its high human blood feeding rate and indoor resting behaviors with high vectorial capacity and adaptability to humans [13]. But reports also showed that the host-seeking behavior of *An. gambiae s.s.* has shifted to outdoor feeding and indoor resting and feed on cattle when humans are not accessible [9, 14, 15]. *An. gambiae s*.*s.* is associated with more humid climates than *An. arabiensis*, which has a greater tolerance for drier environments [16]. *An. arabiensis* feed preferentially on cattle and rest outside of human habitations; it is highly exophilic and zoophagic [8]. Hence, human exposure to *An. arabiensis* bites occurred mostly indoors for LLIN non-users and outdoors for LLIN users. Transmission of malaria relies on the successful development of *Plasmodium* parasites within the mosquitoes, a process termed as sporogony cycle [17]. *An. gambiae s.s*. is highly susceptible to *P. falciparum* infection [18]. The sporogony cycle begins when *Anopheles* mosquitoes ingest human blood containing male and female gametocytes. Sporogony events include gametogenesis, fertilization, zygote differentiation to ookinetes, ookinete passage across the midgut, and forms an oocyst beneath the midgut basal lamina. The oocysts grow, rupture, and release sporozoites, which make their way to the salivary glands of the mosquito. For *P. vivax,* oocysts or sporozoites can be detected at approximately 7–9 or 14–16 days after blood feeding, respectively [19]. Successful mosquito infection rate and oocyst load could be influenced by the parasite density, vector, human factors (the state of gametocyte maturation, proportion of male and female gametocytes) and mosquito factors (age, genetic diversity, and microbiota) [20].

The timeline for sporogony of *P. vivax* is the shortest cycle of human malaria parasites. Within the mosquito abdomen, *P. vivax* sporogony development begins with gametogenesis and the formation of a zygote (within 48 h post-infection), to an ookinete (16–32 h post-infection) and an oocyst (6–9 days post-infection [dpi]), before developing into sporozoites (9–14 dpi). These sporozoites rupture from the abdominal oocyst and migrate through the haemolymph and the thorax to the salivary glands—at this point the mosquito is considered to be infectious [17, 21]. After biting the human, inoculation of the sporozoites into a new human host initiates the parasite exo-erythrocytic and erythrocytic life cycle in humans [22]. The erythrocytic stage of the parasite is responsible for the signs and symptoms in humans. Whilst gametocyte maturation is a long process in *P. falciparum*, infectious *P. vivax* gametocytes appear in the bloodstream within 48 h of blood stage infection [11]. Compared to *P. falciparum*, gametocytes are more commonly observed in *P. vivax* infections, and they appear in blood smears much earlier in an infection [23].

There is a growing concern of *P. vivax* across sub-Saharan Africa [24]. *Plasmodium vivax* represented most malaria infections in traveller to Ethiopia, Eritrea, and Mauritania. Chloroquine or artemisinin-based combinations are the common drugs used against the blood stage *P. vivax* and *P. falciparum* infections. Immature gametocytes are inhibited by artemisinin-based combination therapy (ACT). However, the mature gametocytes of *P. vivax* are relatively insensitive to several anti-malarial drugs [25]. *Plasmodium vivax* is more efficient at being transmitted earlier in the infection and at lower densities than *P. falciparum*, and thus, a greater proportion of individuals infected with *P. vivax* can transmit without detectable gametocytaemia, before becoming ill enough to seek treatment [19, 26]. Furthermore, *P. vivax* appears particularly hard to eliminate [27], principally due to its ability to form dormant liver stages [22].

Hence, innovative tools with proven efficacy on inhibition of *Anopheles* vector survival and disruption of the *Plasmodium* lifecycle are urgently needed. In this context, macrocyclic lactones (ML) endectocides were suggested as a complementary tool to reduce residual malaria transmission [28–30]. ML endectocides are semi-synthetic derivatives of the natural avermectins and milbemycins commonly used in veterinary and human medicine against endo- and ectoparasites. Ivermectin and doramectin are avermectins, moxidectin is milbemycin. The ML endectocides have a wide safety margin and the same mechanism of action as agonists of the glutamate-gated chloride (GluCl) channels in invertebrate postsynaptic neurons and neuromuscular junctions [31].

Studies conducted in humans and livestock showed that ivermectin significantly inhibits the survival of *Anopheles* vectors that fed on treated hosts [28–30, 32–41]. In a recent study, 0.2 mg/kg body weight (BW) of ivermectin and doramectin sustained the insecticidal efficacy on *An. arabiensis* up to 21 days post-treatment of indigenous zebu cattle in Ethiopia and was recommended for malaria vector control via treated livestock [42]. Likewise, a promising insecticidal effect against *An. Gambiae* [43] and *Anopheles Colizzii* [44] was also observed for ivermectin and doramectin in treated cattle*. In-vitro,* ivermectin and doramectin were lethal to *An. arabiensis* at low concentrations (LC50s of 7.9 ppb and 23.9 ppb, respectively), while the lethality of moxidectin was > 100 fold less than for ivermectin [45].

From previous study, after single SC administration, the blood concentration of ivermectin and doramectin remains below lethal dose overtime (after 21 days) in cattle [42]. Sub-lethal concentrations of ivermectin inhibited malaria parasite development in the vector as described in African, South American and Asian *Anopheles* species [28, 46, 47]. The results were promising and would indicate a dual action of ivermectin. Nevertheless, data is scarce on potentially relevant *Anophele*s vectors and *Plasmodium* combinations, namely *An. arabiensis* and *P. vivax* [48]. Hence, the effects of the sublethal concentrations of the MLs on *P. vivax* oocyst development in *An. arabiensis* mosquitoes should be investigated. Therefore, a comparative study assessing i*n-vitro* effects on *An. arabiensis* followed by *ex-vivo* evaluation of ML endectocides (ivermectin, doramectin and moxidectin) sublethal concentrations for their effects on *P. vivax* oocyst development was performed to fill these gaps.

## Methods

### Study design and setting

Experiments were conducted at Jimma University Tropical and Infectious Diseases Research Center (JU-TIDRC) Sekoru district, Jimma zone, Oromia, Ethiopia. Sekoru district is located 240 km southwest of the capital Addis Ababa, Ethiopia (7^o^54′50.0″N, 37^o^25′23.6″E). A parallel study was conducted in three trials at different times between June 2022 and December 2022. Blood samples were collected from *P. vivax* gametocyte infected patients at the health centre nearby JU-TIDRC.

### Ethical statement

This study was performed after receiving ethical approval (Approval Ref. No. IUC-JU/M45/12–2019) by Ethics Review Board (animal care and use ethics committee) of Jimma University, Ethiopia. A formal written consent (in local language, Afan Oromo) for voluntary participation of individual subjects was received. All subjects provided and signed written informed consent.

### Anopheles mosquitoes

The *An. arabiensis* strain originally collected from Bishoftu area colonized at JU-TIDRC was used in the present study. This strain is known to be susceptible to the insecticide classes of pyrethroids, carbamates, organochlorines, and organophosphates [49]. The *An. arabiensis* mosquito colony larvae were reared and maintained as previously described [50]. The larvae were raised on ground Tetramin® fish meal and the adults were provided with 10% (w/v) sugar solution ad libitum at 25 ± 2 °C and 80 ± 10% RH, and 12 h light: 12 h dark photoperiod. Adult female *An. arabiensis* mosquitoes aged 3–5 days were randomly grouped in cups (~ 30 mosquitos per cup) for membrane feeding experiments. Adult female *An. arabiensis* mosquitoes in each group were starved for 6-8 h prior to the membrane feeding experiments.

### Artificial membrane feeding assay

Artificial membrane feeding was conducted using a Hemotek® membrane feeding system (Hemotek Ltd, Accrington, UK) equipped with four Hemotek® feeders. Each feeder contained 1 mL of blood meal with a thin collagen membrane supplied with Hemotek® membrane feeder for mosquito feeding. In both *in-vitro* susceptibility and *ex-vivo* effects on *P. vivax* infection, 1 mL of whole blood spiked with endectocide (ivermectin, doramectin, and moxidectin) at various concentrations was transferred to a Hemotek® feeder maintained at 37 ± 0.1 °C. The feeders were put on top of the paper cups containing the mosquitoes. The mosquitoes were allowed to feed on the blood for a period of 20 min in a dark room. Afterwards, the unfed mosquitoes were removed from the cups by using mouth aspirator and killed by putting them in deep freezer for 20 min. Fully fed mosquitoes were maintained securely in a separate room for 7-days by providing 10% (w/v) sucrose solution soaked in cotton balls twice daily.

### Drug standards and blood meal preparation

Ivermectin, doramectin and moxidectin chemical reference standard powders were obtained from Sigma-Aldrich (Bornem, Belgium). All the standards were stored at ≤  − 15 °C. A fresh working solution (WS) of 40, 20, 4, 2, 0.4, 0.2, and 0.04 µg/mL of the analyte’s concentration were prepared in phosphate buffer solution by serial dilution from the stock solution (1 mg/mL in dimethyl sulfoxide).

For the *in-vitro* study, 1000, 500, 100, 50, 25, 10, 5, and 1 ng/mL concentrations of the endectocide were prepared by transferring 25 µL of 40, 20, 4, 2, 1, 0.4, 0.2, and 0.04 µg/mL of analyte’s working solution, respectively into a tube containing 975 µL cattle whole blood (see Additional file 1: Table S1). Control blood meals consisted of dimethyl sulfoxide diluted (DMSO) in phosphate buffer solution (PBS) to match the concentration found in the highest drug group.

### *In-vitro* susceptibility experiment

Zebu calves with no prior drug history during the last 2 months were used as a blood donor for the *in-vitro* test. Venous blood samples were collected from jugular vein of Zebu calves using 5 mL disposable syringe with a gauge size of the needle between 22 and 23G. on the day of the experiment using EDTA vacutainer tubes. To assess the *in-vitro* susceptibility of *An. arabiensis* mosquitoes to macrocyclic lactone endectocides, various concentrations (1–1000 ng/mL) of the compounds were spiked into cattle blood and fed (for 20 min) to adult female *An. arabiensis* in the treatment group using Hemotek® membrane feeder. The mosquitoes in the control group were fed with blood without the drug MLs. All the unfed and partially fed mosquitoes were gently aspirated, transferred in another cup and killed. Mosquitoes with red distended abdomens were considered as fully fed. Then, the fully fed mosquitoes were provided with 10% (w/v) sugar for 7-days at room temperature. Mosquito survival was monitored for 7 days. Every day, dead mosquitoes were removed and recorded. On day 7, all remaining mosquitoes were frozen and counted as alive.

### *Plasmodium vivax* infected patients volunteer recruitment

Recruitment of *P. vivax* patients was conducted from October to December 2022 in Deneba health center of Sekoru district. All study participants signed the informed consent for voluntary participation after a brief explanation. *Plasmodium vivax* gametocyte infected patients were diagnosed using light microscopy of Giemsa-stained blood samples. The patients infected with *P. vivax* were consented if they met the following inclusion criteria: age > 18 years, history of previous malaria episodes, no signs of severe disease and no previous antimalarial treatment history during the last 4 weeks. Venous blood (3 ml) was collected with a 5 mL gauge syringe into EDTA vacutainer tubes. After blood collection, the patients were treated for *P. vivax* infection following the standard treatment guidelines.

### *Ex-vivo P. vivax* infection of mosquitoes

The *ex-vivo* evaluation of the endectocides effects on *P. vivax* oocyst development in* An. arabiensis* mosquito was performed as previously described [28, 46] with some modifications. After enrollment, *P. vivax*-infected venous blood (3 mL) was drawn on site and pooled for the experiments at JU-TIDRC. The blood samples collection and transport of was based on the method previously reported [51, 52]. Initially the airtight thermos flask was validated for maintaining the temperature at 37 ± 2 °C during transport. Accordingly, the fresh patient’s blood samples in EDTA vacutainer tubes were put in airtight thermos flask maintained at 37 °C in a water. The tubes were positioned to float on top of the flask and tightened very well. Then, it was taken to the nearby JU-TIDRC and transferred into the tubes and spiked with the endectocides followed by immediate mosquito feeding. The control groups were fed with untreated infected blood. The *P. vivax* infection experiment in mosquitoes was conducted in a separate and secured laboratory set-up at room temperature (25–27 °C) and a 12:12 light: dark cycle. The experiment was conducted at the Arthropod Containment Level-1 (ACL-1) Lab in the JU-TIDRC with restricted human access throughout the experiment. *Anopheles arabiensis* mosquitoes were allowed to feed on *P. vivax*-infected venous blood spiked with the endectocides at LC_25_, LC_5_ concentrations (see in Additional file 1: Table S2) and the control. An average of 25 mosquitoes per cup (previously grouped and starved for 6-8 h) were put under a Hemotek® membrane feeder device (at 37˚C) containing 1 mL of the endectocide with the *P. vivax* gametocyte infected blood for 30 min. Fully engorged mosquitoes were left in the paper cups, and securely maintained at mosquito infection insectary room and provided with 10% (w/v) sugar solution ad libitum for 7-days. On day 7, all the surviving mosquitoes were dissected and examined. The study was conducted in three replicates.

### Mosquito dissection and oocyst examination

The dissection and oocyst examination was based on the previous described method by Ouedraogo et al*.* [53]. After 7-days post-feeding, the mosquitoes were killed by placing them in the freezer at − 20 °C for 10 min. Then, for mosquito dissection, each mosquito was held on a non-frosted slide under a dissection stereo microscope. Mosquito dissection was conducted by pulling the abdomen apart with forceps until the midgut was exposed. The midgut stained with 0.5% (w/v) Mercurochrome (Sigma-Aldrich, USA) solution for 10 min followed by covering with coverslips. *Plasmodium* oocysts were examined at 400-fold magnification and enumerated using compound microscope (Optical Microscopy, Olympus, Germany). Infection prevalence was expressed as percentage of mosquitoes with at least one oocyst.

### Statistical analysis

The statistical analysis for both mosquito mortality and oocyst prevalence was based on a generalized mixed model with binomially distributed error term using SAS version 9.4 (SAS Institute, Inc., USA). Mortality at day 7 is the response variable where the drug dose was considered as a continuous fixed effects factor and replicate number as a random effects factor. The effect of endectocides LC_25_ and LC_5_ on the presence and abundance of *P. vivax* oocyst was analysed statistically. The variables include the proportion of mosquitoes infected with *P. vivax* oocyst (oocyst prevalence) and the number of oocysts per mosquito midgut (oocyst intensity). The relationship between the sublethal doses and the endectocides on the binary response variables of oocyst prevalence (absent or present) was analysed from the three replicates. Results are expressed in terms of the odds ratio for one log unity increase in concentration, and further interpreted by visualization of the dose response curve.

The sub-lethal concentrations (LC_50_, LC_25_ and LC_5_) were estimated based using logistic regression together with probit analysis. The oocysts intensity in treatment and control groups were compared using the nonparametric Kruskal Wallis test and the Mann–Whitney U test for single comparisons.

## Results

### *In-vitro* susceptibility and LC25 and LC5 determination in *Anopheles arabiensis*

**Fig. 1:**
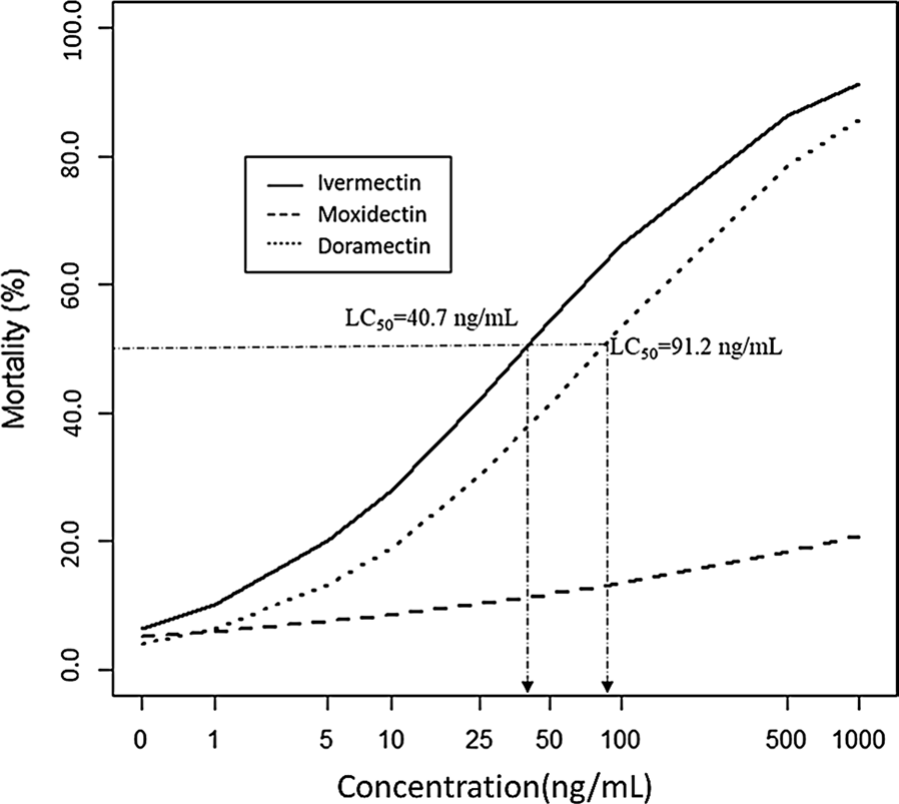
*Anopheles arabiensis* mosquito mortality (in percentage) including the biological death after ingestion of cattle blood spiked with different concentrations (in ng/mL) of ivermectin, doramectin and moxidectin

In the present study, a total of 1,417 female *An. arabiensis* mosquitoes imbibed the three macrocyclic lactone (ML) endectocides in 8 different concentrations (1000, 500, 100, 50, 25, 10, 5, 1 ng/mL) in cattle to estimate lethal concentrations for each compound. The mortality of *An. arabiensis* within 7-days of post-feeding showed that there were significant dose–response curves for the three drugs (P < 0.001) (Fig. 1). At the lower doses, a significant (P < 0.001) dose-dependent insecticidal effect was obtained in both ivermectin and doramectin. The highest mortality was found for ivermectin, while the lowest was registered for moxidectin. The odds ratio for one log unit increases in concentration of 2.07 (95% CI: [1.85;2.33]) for ivermectin, 2.05 (95% CI: [1.83;2.31]) for doramectin, and 1.26 (95% CI: [1.12;2.41]) for moxidectin, and all differed significantly from 1 (P < 0.001).

The LC_50_, the concentration that kills 50% of the mosquitoes, was determined from the mortality within 7-days of post-feeding using the logistic regression results. LC_50_ (ng/mL) of 40.7 (95% CI: [36.4;45.8]), 91.2 (95% CI: [81.4;102.8]) and 5128.6 (95% CI: [4625.6;9790.3]) were obtained, respectively, for ivermectin, doramectin and moxidectin. Accordingly, the LC_25_ and LC_5_ sub-lethal concentrations were determined as described below (Table 1).

**Table 1:** Ivermectin, doramectin, moxidectin LC_50_*,* LC_25_ and LC_5_ after Hemotek® membrane feeding of animal blood reconstituted with different dose or concentration (in ng/mL)) of ivermectin, doramectin and moxidectin

Sub-lethal concentrations	Ivermectin	Doramectin	Moxidectin
LC_50_	40.7[36.4–45.8]	91.2[81.4–102.8]	5128.6[4625.6–9790.3]
LC_25_	7.1[6.3–8.0]	20.0[17.8–22.5]	794.3[716.4–1516.3]
LC_5_	0.6[0.5- 0.7]	1.8[1.6–2.0]	53.7[48.4–102.5]

Key: The number in bracket indicates the 95% CI; LC_50_, LC_25_ and LC_5_, corresponds to the concentrations that kill 50%, 25% and 5% of the mosquitoes respectively

**Fig. 2:**
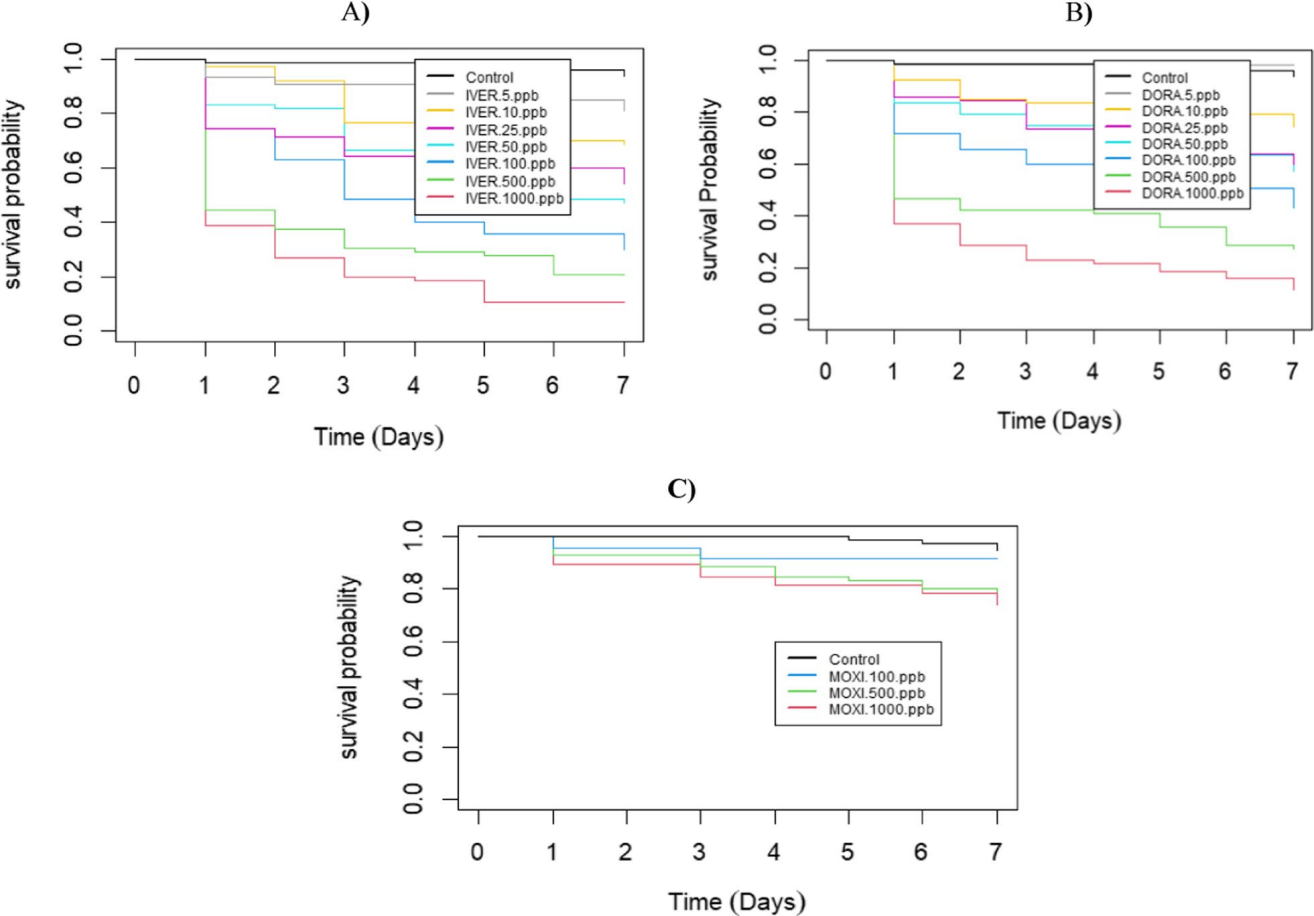
Kaplan–Meier survival curves showing the survival probability of *Anopheles arabiensis* mosquitoes as a function of time after feeding on cattle blood spiked with different concentration (ng/ml)) of the ML endectocides ivermectin (IVER) (**A**), doramectin (DORA) (**B**) and moxidectin (**C**)

During the 7-days of post-blood feeding, the survival probability was significantly different from the control at all concentrations above 10 ppb in both ivermectin and doramectin groups. The survival probability of mosquitoes dropped by more than 30% starting from the day 1 post-feeding of ivermectin and doramectin except in the lower concentrations (Fig. 2). The insecticidal effects induced by moxidectin was not significantly different from the control.

### *Ex-vivo* evaluation of endectocides on *P. vivax* oocyst development

**Fig. 3:**
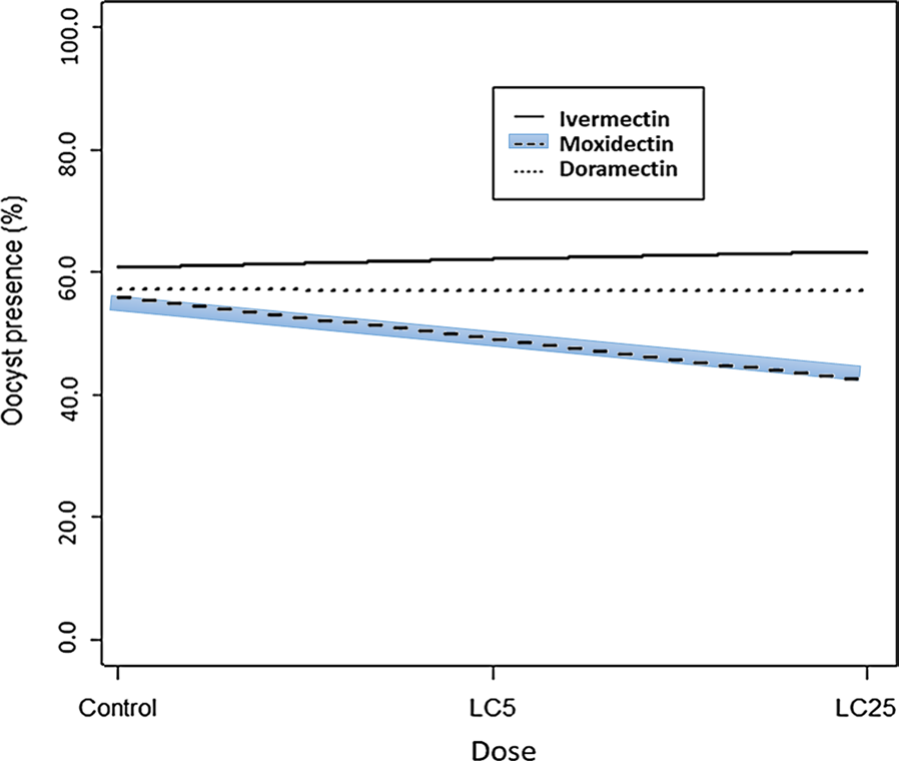
*Plasmodium vivax* oocyst prevalence (in percentage) versus dose administered in *Anopheles arabiensis* mosquitoes in the ivermectin, moxidectin, doramectin treated and control groups. Control refers to those administered with *P. vivax* infected blood without drug

**Fig. 4:**
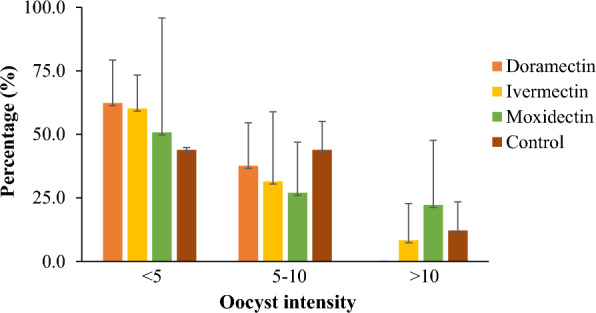
Oocyst intensity among *Plasmodium vivax* infected *Anopheles arabiensis* mosquitoes treated with doramectin, ivermectin, moxidectin and control groups

A total of 13 *P. vivax* infected gametocyte carrier patients fulfilling the inclusion criteria were enrolled in the present study. The oocyst prevalence did not change significantly as a function of dose, i.e., control, LC_5_ and LC_25_ (Fig. 3), with odds ratios equal to 1.06 (95% CI: [0.65;1.72]), 0.99 (95% CI: [0.64;1.56]) and 0.76 (95% CI: [0.47;1.22]) for ivermectin, doramectin and moxidectin. The oocyst intensity in the treatment groups were also not significantly (p > 0.05) different from each other and from control (Fig. 4).

## Discussion

*Plasmodium vivax* is more efficient at being transmitted at an earlier stage and a greater proportion of individuals infected with *P. vivax* can transmit without detectable gametocytaemia. *Plasmodium vivax* infected persons are more likely to transmit before seeking treatment, compared to *P. falciparum* [26]. In addition to killing the mosquitoes, reducing the infectivity of zoophagic mosquito population by the sublethal concentrations of the endectocides in treated human or livestock may prevent malaria transmission[54]. In Ethiopia, ivermectin mass drug administration (MDAs) are being performed for onchocerciasis and scabies [55]. Ivermectin MDA to humans also has an insecticidal effect on *Anopheles* mosquitoes and may contribute to the suppression of malaria transmission. Livestock, mainly cattle, serve as adequate blood source for *An. arabiensis* allowing vector populations to persist [14]. Previous studies showed that treatment of livestock with the subcutaneous formulations of the endectocides ivermectin and doramectin led to an insecticidal effect up to 3 weeks for *An. arabiensis* mosquitoes [42, 56] and on repeated treatment in humans [39]. A similar evidence is also available from previous *in-vitro* study of ivermectin on *An. arabiensis* [57]. However, the effects of the endectocides on *P. vivax* is not well investigated. Doramectin and moxidectin are the other endectocides recently introduced into African market for use in livestock. Therefore, this study is the first to report the comparative evaluation of the sub-lethal concentration of the ML endectocides effects on *P. vivax* oocyst development in *An. arabiensis* on concomitant ingestion with the parasite.

In the present study, a significant mortality of *An. arabiensis* was induced *in-vitro* by both ivermectin and doramectin, with ivermectin having the largest effect and moxidectin the least. A similar finding was reported previously [34, 42, 43, 45]. No effect of pig moxidectin treatment [58] or cattle moxidectin treatment was also previously reported [43, 59]. A concentration-dependent insecticidal effect was observed in both ivermectin and doramectin. At lower concentrations there was a delayed mortality effect. This may be attributed to the fact that endectocides reduce the locomotion of the mosquitoes due to their action on glutamate-gated chloride channels in neuromuscular tissues which increases their chance of death or reducing the vector capacity regardless of the mortality [60]. As a result, especially on human mass drug administration, the sub-lethal concentrations of ivermectin and doramectin could potentially reduce malaria transmission by preventing the vector from taking another blood meal. A delay in re-feeding would reduce likelihood of future infectious bites to the human population [46, 47].

No evidence of reduction in *P. vivax* oocyst prevalence in *An. arabiensis* was observed when ivermectin (LC_25_ = 7.1 ng/mL and LC_5_ = 0.6 ng/mL), doramectin (LC_25_ = 20.0 ng/mL and LC_5_ = 1.8 ng/mL), or moxidectin (LC_25_ = 794.3 ng/mL and LC_5_ = 53.7 ng/mL) sub-lethal concentration were ingested compared to the control groups. Among the infected mosquitoes, the *P. vivax* oocyst intensity in the treatment groups was not significantly (P > 0.05) different from the control groups. Therefore, the sublethal concentrations (LC_25_ and LC_5_) of ivermectin, doramectin and moxidectin do not seem to induce relevant effects on *P. vivax* oocyst development on concomitant ingestion with the parasite in *An. arabiensis*. A similar finding was reported for ivermectin on *P. vivax* oocyst development in* An. darlingi* on experiments [28, 46] with a slight increased LC_50_ values. In addition, the species related factor, the experiment setup and the method used in the present study contributed to the observed difference. The findings in the present study showed the effects of endectocides at lower concentrations on concomitant administration with *P. vivax*-infected blood to mosquitoes. Nevertheless, the effects on non-concomitant exposures to endectocides and *P. vivax*-infected blood in mosquitoes were not included. This is considered as a limitation. Hence, future research could investigate the effect of the higher concentrations and metabolites. It takes 7–10 days for the *P. vivax* oocyst to develop from the gametocyte stage in the mosquito [61]. The action of endectocides on glutamate-gated chloride channels in neuronal and neuromuscular tissues of invertebrates [60], such as *Anopheles* mosquitoes, was suggested for their insecticidal effects [42]. However, this molecular target is absent in *Plasmodium* species which might explain that the endectocides lack effects on *P. vivax* oocyst development in the present study.

The mode of action of ivermectin MDA is most likely through a reduction in daily survivorship/longevity of adult females. According to the Ross-Macdonald model, reductions in survivorship/longevity have substantial impacts on vectorial capacity, suggesting ivermectin MDA could substantially reduce malaria transmission [62]. In a previous study [42], the endectocides ivermectin and doramectin significantly shortened the lifespan of *An. arabiensis* (*in-vivo)* in treated cattle, as was observed in the present *in-vitro* membrane feeding assay. Glutamate-gated chloride channels (GluCl) are abundantly found in *Anopheles* mosquitoes. The insecticidal effects of ML endectocides were mainly due to the pseudo-irreversible effect in opening these channels to the influx of chloride ions. In the same mechanism, the sub-lethal concentrations of ivermectin and doramectin potentially reduce the daily probability of mosquitoes feeding on humans by affecting the functionality of the mosquito’s locomotion and their re-feeding potential [43, 59].

## Conclusion

In conclusion, the endectocides ivermectin and doramectin significantly reduced *An. arabiensis* survival demonstrating their potential for malaria vector control. No direct effect of ML endectocides on *P. vivax* infection in *An. arabiensis* mosquitoes was observed using the LC_25_ and LC_5_ values. the effects of ivermectin and doramectin on malaria parasite transmission are most likely attributed to their indirect effect on *An. arabiensis* by reducing adult lifespan thereby causing their death before completing the sporogony cycle.

### Supplementary Information

**Additional file 1: Table S1.** Preparation of ivermectin, doramectin, and moxidectin concentrations (1-1000ng/mL) in spiked cattle blood. **Table S2.** Preparation of ivermectin, doramectin, and moxidectin sublethal concentrations (LC_25_ and LC_5_) in spiked *Plasmodium vivax* infected blood. **Annex I.** Malaria Patient’s Information Sheet. **Annex II.** Study participant informed Consent.

## Data Availability

The data presented in this study are available on request from the corresponding author.
