# Supplementary material for: In-vitro susceptibility and ex-vivo evaluation of macrocyclic lactone endectocides sub-lethal concentrations against Plasmodium vivax oocyst development in Anopheles arabiensis
Source: Malar J. 2024 Jan 18;23:26. doi: 10.1186/s12936-024-04845-x (PMC10797976; doi:10.1186/s12936-024-04845-x)
Supplement: Supplementary file 1 — Additional file 1: Table S1. Preparation of ivermectin, doramectin, and moxidectin concentrations (1-1000ng/mL) in spiked cattle blood. Table S2. Preparation of ivermectin, doramectin, and moxidectin sublethal concentrations (LC25 and LC5) in spiked Plasmodium vivax infected blood. Annex I. Malaria Patient’s Information Sheet. Annex II. Study participant informed Consent. [file 12936_2024_4845_MOESM1_ESM.docx]

**Additional file 1**

**Table S1.** Preparation of ivermectin, doramectin, and moxidectin concentrations (1-1000ng/mL) in spiked cattle blood.

| Concentration(ng/mL) | Volume of working solution (µL) | Cattle blood (µL) |
| --- | --- | --- |
| 1000 | 25µL of 40µg/mL | 975µL |
| 500 | 25µL of 20µg/mL | 975µL |
| 100 | 25µL of 4µg/mL | 975µL |
| 50 | 25µL of 2µg/mL | 975µL |
| 25 | 25µL of 1µg/mL | 975µL |
| 10 | 25µL of 0.4µg/mL | 975µL |
| 5 | 25µL of 0.2µg/mL | 975µL |
| 1 | 25µL of 0.04µg/mL | 975µL |

Key: nanogram per milliliter, ng/mL; microgram per milliliter, µg/mL; microliter, µL

**Table S2.** Preparation of ivermectin, doramectin, and moxidectin sublethal concentrations (LC_25_ and LC_5_) in spiked *Plasmodium vivax* infected blood.

| **Drug** |  | **Sublethal concentrations Concentrations(ng/mL)** | | **Volume of working solution (µL)** | **Volume of infected blood (µL)** |
| --- | --- | --- | --- | --- | --- |
| Ivermectin |  | LC_25_ | 7.1 | 24µL of 300ng/mL | 976µL |
|  |  | LC_5_ | 0.6 | 24µL of 25ng/mL | 976µL |
| Doramectin |  | LC_25_ | 20.0 | 20µL of 1000ng/mL | 980µL |
|  |  | LC_5_ | 1.8 | 36µL of 50ng/mL | 965µL |
| Moxidectin |  | LC_25_ | 794.3 | 20µL of 40µg/mL | 980µL |
|  |  | LC_5_ | 53.7 | 27µL of 2µg/mL | 973µL |

Key: nanogram per milliliter, ng/mL; microgram per milliliter, µg/mL; microliter, µL

**Annex I: Malaria Patient’s Information Sheet**

**Organization:** Jimma University, Institute of Health

**Name of Sponsor:** Jimma University and Ghent University

**Research Title:**  *In-vitro* susceptibility and *ex-vivo* evaluation of macrocyclic lactone endectocides sub-lethal concentrations against *Plasmodium vivax* oocyst development in *Anopheles arabiensis*.

Investigators:

- Mr. Gemechu Zeleke

- Prof. Sultan Suleman

- Prof. Dr. Mathias Devreese

**Purpose of the research**

We are undertaking PhD research at Tropical and Infectious Disease Research Center (TIDRC) of Jimma University and in the surrounding Jimma zone within collaborative framework of Jimma University and Ghent University. We are doing this research using malaria patients. We would like to investigate the effect of MLs on the malaria parasite P. vivax in *An. arabiensis* mosquitoes. We are now giving you information and invite you to voluntarily participate in this research. Before you decide, you can talk to anyone and feel free, comfortable, and rationally decide to participate in this research. There may be some words that you do not understand. Please ask and we will take time to explain. If you have questions later, you can ask freely.

**What will we do?**

We will take a maximum of 3mL of veinous blood from your arm for the study and this will be done only once. Taking blood sample might be a slight uncomfortable and may induce mild pain/swelling at the injection site that will disappear within a minute. We use only sterile and new needles and syringes to prevent the risk of infection. The sample of veinous blood will be taken by a well trained and experienced laboratory technologist. This reduces unwanted pain-full and stress conditions in the procedure. You will be provided will feed antimalarial drugs continuous follow-up for improvement of the malaria disease symptoms and effective curative therapy.

**Potential benefits**

In addition to getting effective diagnosis and curative therapy, your participation will benefit the community in reducing malaria transmission. The community will benefit from the research output. in the future and we will hopefully reduce and finally eliminate malaria transmission.

**Potential risks**

Slight pain and discomfort at the site of injection (which disappears or resolves within a minute) that you will feel during blood sample collection.

**Confidentiality**

The collected information will be kept confidential. The data will be collected by coding and only the researchers will be able to see it. It will not be shared with or given to anyone except those who have access to the information, such as researchers and supervisors.

**Whom to contact**

If you have any questions about this research, you can contact the following individuals:

Mr. Gemechu Zeleke, Jimma university, Ethiopia, Mobile phone: +251-922421574, E mail: zelekecarii@yahoo.com

Dr. Sultan Suleman, Jimma university, Ethiopia, Mobile Phone: +251-911-74-23-54, E-mail: sultan.sulemanl@gmail.com

**Annex II: Study participant informed Consent**

**Organization:** Jimma University, Institute of Health

**Name of Sponsor:** Jimma University and Ghent University

Your signature below confirms your willingness to voluntarily participate in this research.

I have read the foregoing information regarding this research, or it has been read and explained to me. I have had the opportunity to ask questions about it and all the questions that I have asked has been answered to my satisfaction. I understand that I am free to choose to be in this study or that saying “NO” will have no effect on me. I understood and agreed to donate 3 ml veinous blood samples only once in this study. I understand that this may be slightly uncomfortable. I also understand that while taking blood by syringe these do not cause infection. I also give permission to share the facts collected through this study, without stating my name with other studies.

Name of participant __________________ If illiterate

Signature ___________________, Thumb print

Date ___________________________ Day/month/year

**Statement by the researcher/person taking consent**

I confirm that the participant was given an opportunity to ask questions about the study, and all the questions asked by the participant have been answered/explained adequately and to the best of my ability. I confirm that the individual has not been coerced into giving consent, and the consent has been given freely and voluntarily.

 Name of Researcher/person taking the consent________________________

Signature of Researcher /person taking the consent__________________________

Date ___________________________ Day/month/year

**Annex II: Waligaltee/Ragaa fedhitiin qoranno kana irratti Hirmachisuu**

**Maqaa wajira:** Universitii Jimmaa fi Universitii Ghent

**Qoranno kana kan maallaqan degare:** Universitii Jimmaa fi Universitii Ghent

Odeefannoon qorannoo armaan olitti caqafame naaf dubifameera. Akkasumas gaafiin gaafadheefis deebiif ibsi quubsaan naaf kennameera. Dirqama ykn dhibbaa nam-tokko malee qoranno kana irratti fedhii kootiin qofaa hirmaachisuu ykn dhiisuu akkan danada’u naan beeka. Kannaf qorannoo kana iratti hirmachuun koo fedhii kootiin qofaa akka ta’e nan mirkaneessa. Qoranno kana irraatti dhigni koo hangi 3ml ta’u yeroo tokko qofaaf akkan kennu nan beeka. Kun immoo xiqoo nattii toluu baatu illee, fedhiii kootiin eeyyamuu koo nanmirkaneesa. Ragaan fudhatan kun maqaa koo otoo hin caqasiin qorannoowan keessatti akka fayyadaman nan-eeyyama. Kanaaf mallaton koo kana gadii fedhiitiin qorannoo kana irratti horii koo hirmachisuu koo ibsa.

Maqaa abbaa horii: _____________________ Kan hin baranne yoo ta’e

Mallato: _____________________ Mallattoo qubaa

Guyya/Ji’a/Bara: ___________________

Hirmaataan qoranaa kaatiif carraan waan hin hubanneratti gaaaffii gafachuu argachuusaa/shee fi gaaffi ka’e irratti deebii qubsaa ta’e hanga dandeetiikooti kennameera. kanaaf hirmaataan kun fedha isaatiin dhiibbaa tokko malee qorannoo kana irratti hirmachusaa/ishe nan mirkaneessa.

Maqaa qorataa__________________

Mallattoo ___________________

Guyya: ___________________ Guyya/Ji’a/Bara
